# Druggists

**Published:** 1860-11

**Authors:** 


**Druggists.**

We find in the transactions of the American Pharmaceutical Association, which met in New York on the 11th of September last, the passage of a resolution requiring the appointment of a Committee to report to the next meeting of the Association a plan for the regulation of the sale of poisons by Apothecaries in the United States. The committee of three appointed under the resolution, is composed of the following gentlemen: Sam'l W. Colcord, Boston; William Proctor, jr., Philadelphia; W. J. M. Gordon, Cincinnati.

Now we think all this is well enough, as far as it goes, but until Druggists and Apothecaries deliberate upon the subject of vending patent medicines and secret nostrums, they have not touched the main question connected with the sale of injurious substances, and with the welfare of the commuty. The number of persons destroyed by known poisons, by design and accident, are small compared with those that suffer, directly and indirectly, by patented unknown substances. The physician appeals in vain to the people who swallow the highly lauded pill, syrup, or tincture, for the arrest of the evil, because they misinterpret his object. The appeal to druggists is unheeded, because from the sale of such articles much of their profit is derived. Pharmaceutists know that the object of physicians, in opposing the use of nostrums, is a good one—that their business is increased by the general use of patent medicines, and that fortunes are filched from the pockets of credulous invalids, by deceptive and designing charlatans. The knowledge of these things, it is hoped, will lead to some movement of the Association in this direction.

While the dry-goods-man may sell a flimsy article at a remunerative price, to a customer, who fails to get the worth of his money, and feel that no serious injury has been done, the druggist's business is a more responsible one, his mission higher. His commodities affect the health, the life of his customers.

We have, in Atlanta, four Druggists in successful business, and we are pleased to see the Patent Medicine shelves becoming less and less crowded, and their contents more and more dusty; not on account of the increase of sales, but from the decrease in the supply of the abominable stuff. We are pleased to see an amount of trade in the sale of pure drugs and medicines sufficient fully to sustain our druggists, even at the reasonable prices which they ask; and as they lessen the weight upon their patent medicine shelves by failing to purchase, or receive, as agents, such articles, we expect to see physicians in every direction crowding these houses for their supplies of drugs. Their reliability as pharmaceutists, uprightness in business transactions, and their extensive assortment and reasonable prices of drugs, will secure them the patronage of physicians in convenient portions of this and adjoining States.

---

☞ The climate in Minnesota is said to have a very salutary influence on Consumptives, when no material disorganization of the lungs exists.

---

☞ The number of births last year in Philadelphia, was 14,832 of there 7,659 were males, and 7,163 females. The deaths during that year amounted to 9,742, of males, 5,160, of females 4,582.

---

☞ Dr. H. P. Dwees, of New York, has invented an instrument for the local application of anæsthetic and stimulating vapors, designed for the application of irritating substances as well as soothing and anæsthetic.

---

☞ Dr. H. F. Campbell, of Augusta, Ga., has invented a plug for the Uterian Spectulim, which, it is said, prevents the difficulty and pain sometimes found in the introduction.

